# Supplementary figures and images for: Defining substrate requirements for cleavage of farnesylated prelamin A by the integral membrane zinc metalloprotease ZMPSTE24
Source: PLoS One. 2020 Dec 14;15(12):e0239269. doi: 10.1371/journal.pone.0239269 (PMC7735620; doi:10.1371/journal.pone.0239269)

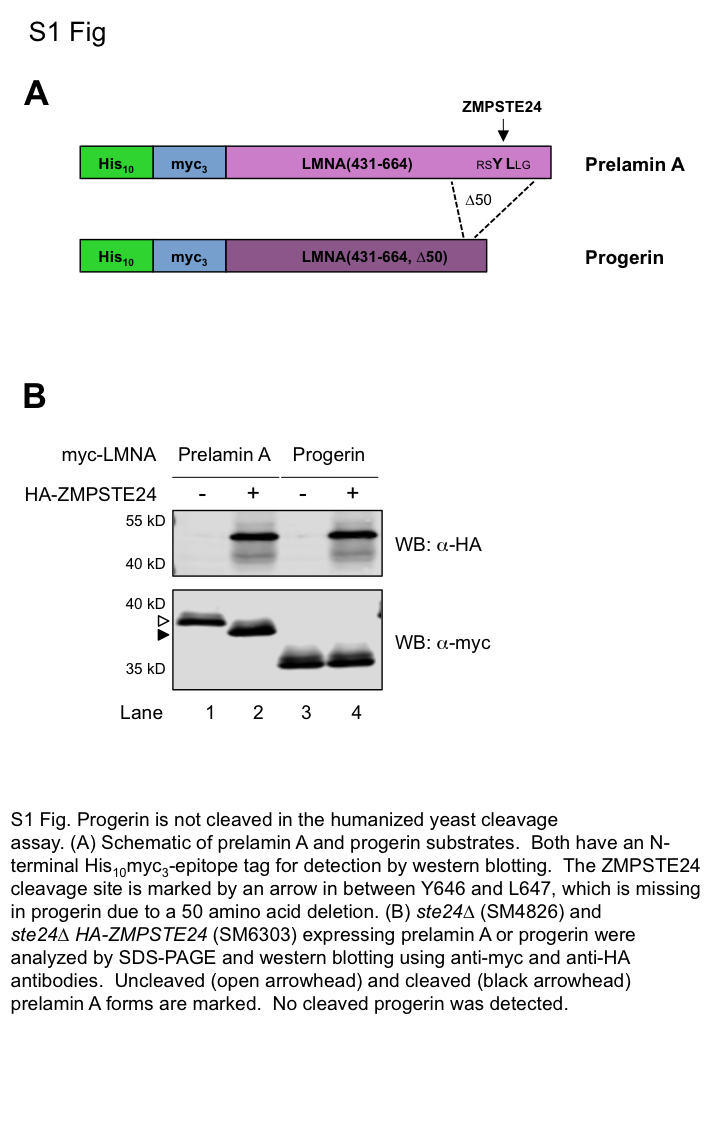

Supplement: S1 Fig — (A) Schematic of prelamin A and progerin substrates. Both have an N-terminal His10myc3-epitope tag for detection by western blotting. The ZMPSTE24 cleavage site is marked by an arrow in between Y646 and L647, which is missing in progerin due to a 50 amino acid deletion. (B) ste24Δ (SM4826) and ste24Δ HA-ZMPSTE24 (SM6303) expressing prelamin A or progerin were analyzed by SDS-PAGE and western blotting using anti-myc and anti-HA antibodies. Uncleaved (open arrowhead) and cleaved (black arrowhead) prelamin A forms are marked. No cleaved progerin was detected. (TIFF) [file pone.0239269.s001.tiff]

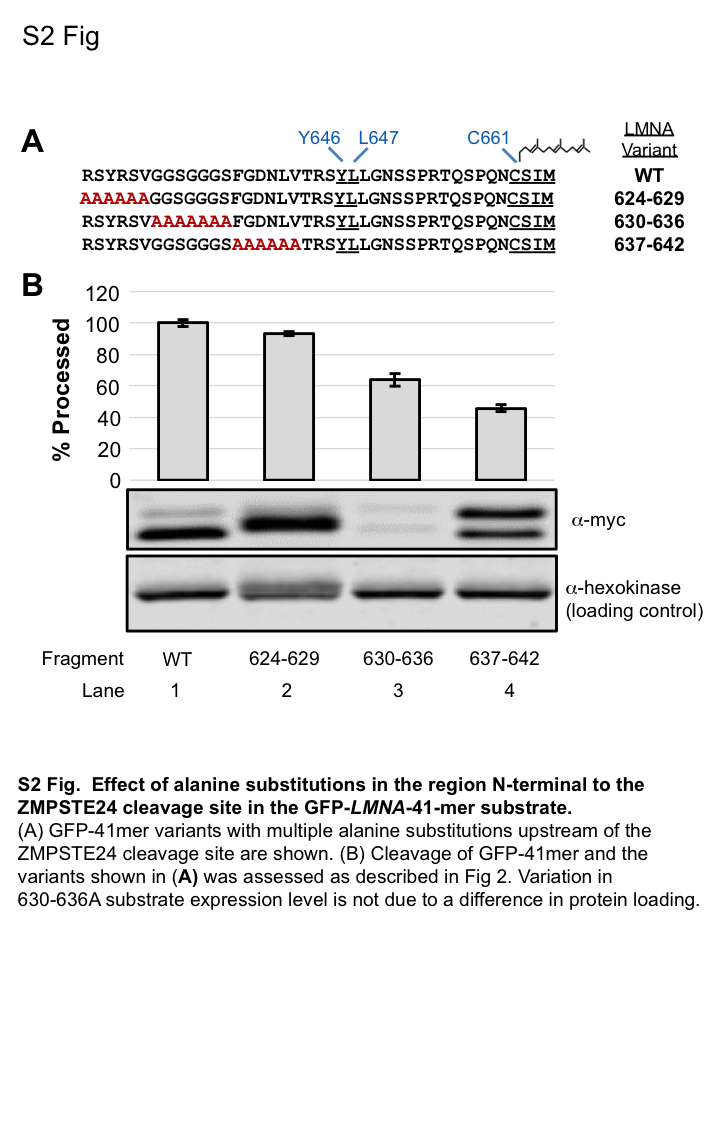

Supplement: S2 Fig — (A) GFP-41mer variants with multiple alanine substitutions upstream of the ZMPSTE24 cleavage site are shown. (B) Cleavage of GFP-41mer and the variants shown in (A) was assessed as described in Fig 2. Variation in 630-636A substrate expression level is not due to a difference in protein loading. (TIFF) [file pone.0239269.s002.tiff]
