# Supplementary material for: Defining substrate requirements for cleavage of farnesylated prelamin A by the integral membrane zinc metalloprotease ZMPSTE24
Source: PLoS One. 2020 Dec 14;15(12):e0239269. doi: 10.1371/journal.pone.0239269 (PMC7735620; doi:10.1371/journal.pone.0239269)
Supplement: S1 Raw Images — (PDF) [file pone.0239269.s003.pdf]

Fig. 2B

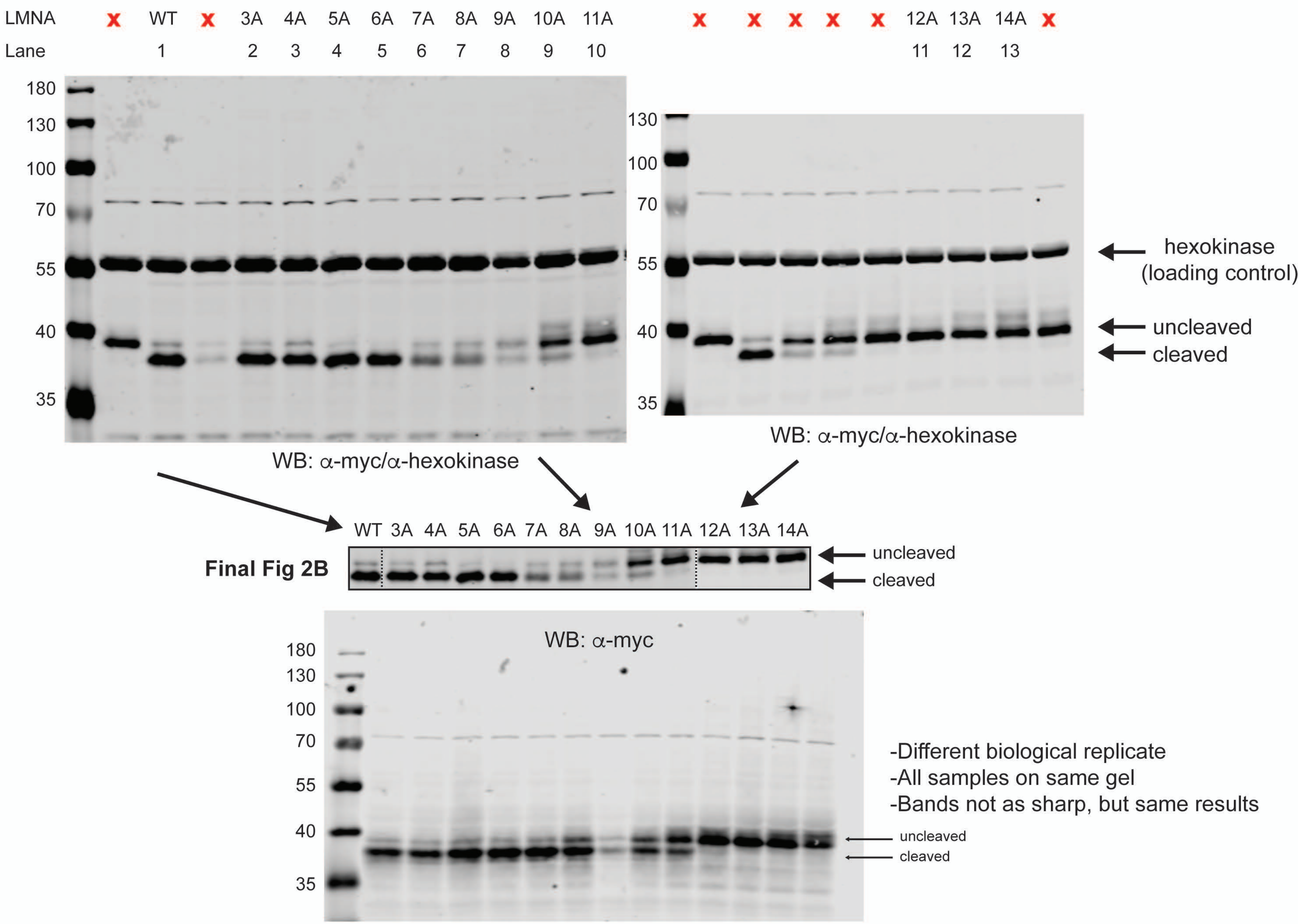

- SDS detergent lysates from strain SM6303 (*ste24Δ HA-ZMPSTE24*) transformed with myc-prelamin A plasmids were resolved by 10% SDS-PAGE, transferred to nitrocellulose (Biorad TransBlot Turbo), and proteins were detected using anti-myc monoclonal antibody and rabbit anti-hexokinase polyclonal antibody (described in M&Ms). Proteins were detected using Licor secondary antibodies and visualized using the Odyssey imaging platform (Licor). Some LMNA mutants show lower steady-state levels (lanes 6-8). This has also been observed for other LMNA mutants.

Fig. 3B

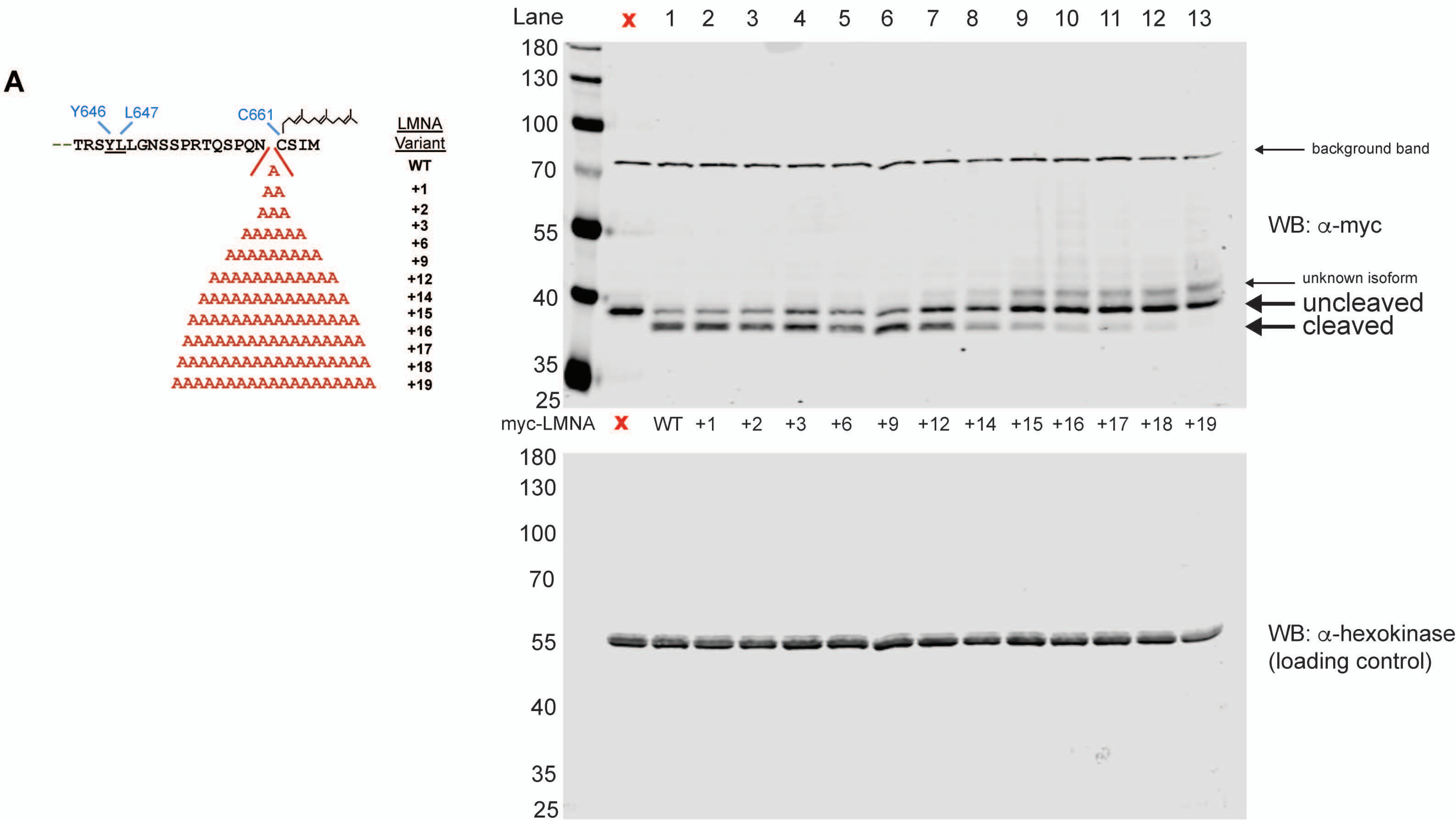

- SDS detergent lysates from strain SM6303 (*ste24Δ HA-ZMPSTE24*) transformed with myc-prelamin A plasmids were resolved by 10% SDS-PAGE, transferred to nitrocellulose (Biorad TransBlot Turbo), and proteins were detected using anti-myc monoclonal antibody and anti-hexokinase polyclonal antibody (described in M&Ms). Proteins were detected using Licor anti-mouse and anti-rabbit secondary antibodies and visualized using the Odyssey imaging platform (Licor). The longer alanine extensions (+14-+19) show a slower migrating species of unknown origin.

Fig. 4B

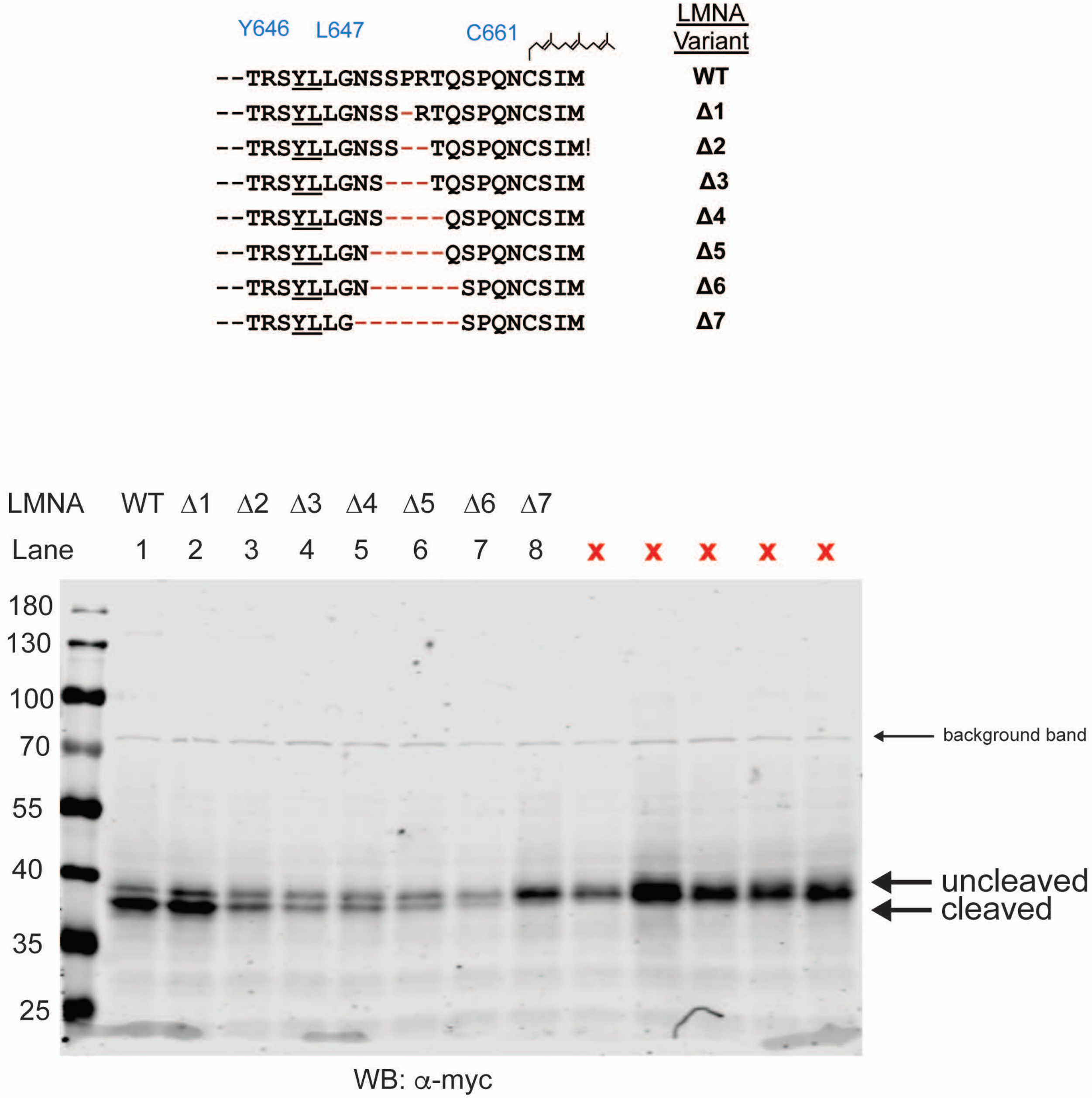

- SDS detergent lysates from strain SM6303 (*ste24Δ HA-ZMPSTE24*) transformed with myc-prelamin A plasmids were resolved by 10% SDS-PAGE, transferred to nitrocellulose (Biorad TransBlot Turbo), and proteins were detected using anti-myc monoclonal antibody (described in M&Ms). Proteins were detected using Licor anti-mouse secondary antibodies and visualized using the Odyssey imaging platform (Licor). Some LMNA mutants show lower steady-state levels (lanes 3-7). This has also been observed in Figure 6B.

Fig. 5B

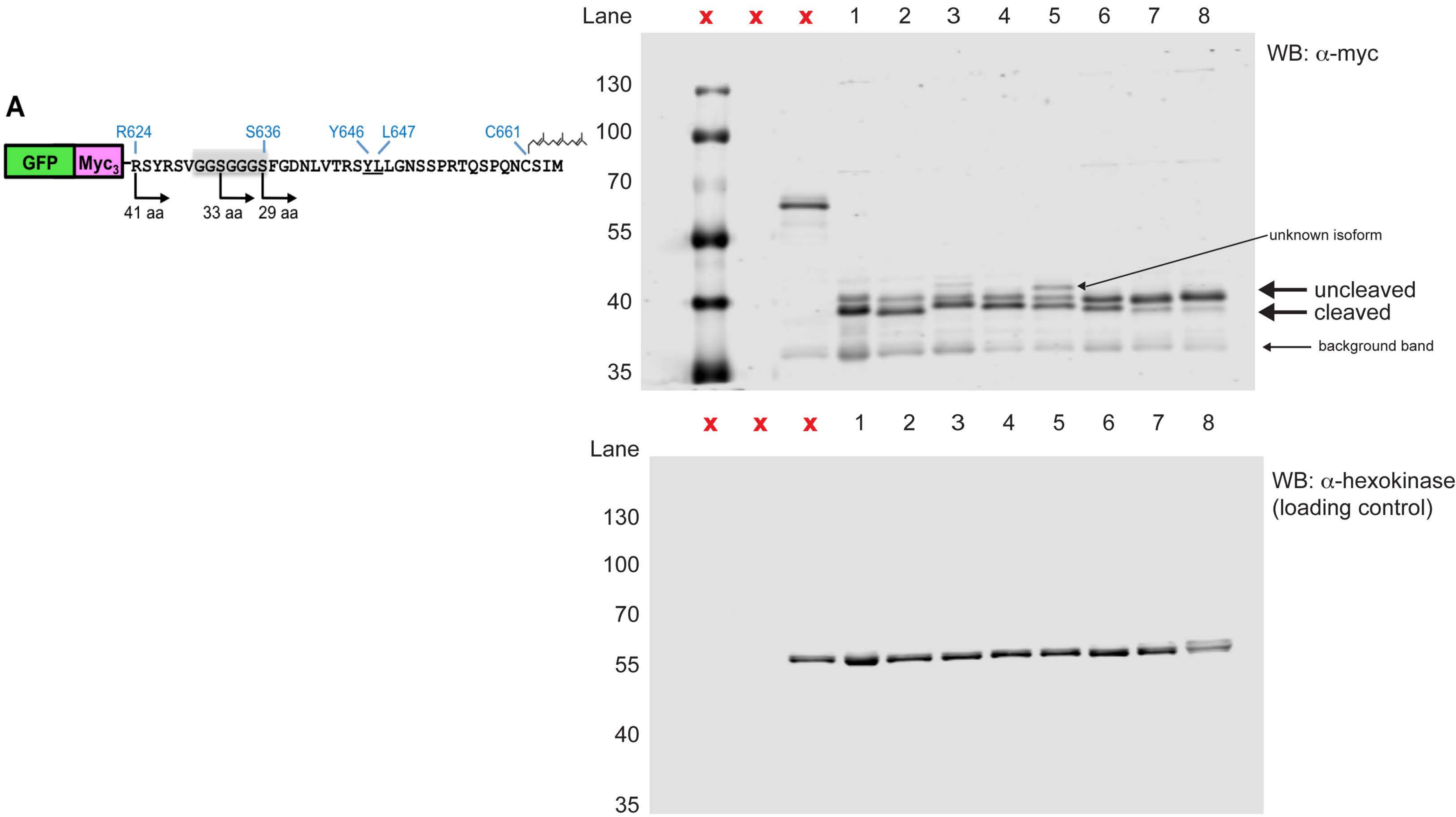

- SDS detergent lysates from strain SM6303 (*ste24Δ HA-ZMPSTE24*) transformed with myc-prelamin A plasmids were resolved by 10% SDS-PAGE, transferred to nitrocellulose (Biorad TransBlot Turbo), and proteins were detected using anti-myc monoclonal antibody and anti-hexokinase polyclonal antibody (described in M&Ms). Proteins were detected using Licor anti-mouse and anti-rabbit secondary antibodies and visualized using the Odyssey imaging platform (Licor). The longer alanine extensions (+14-+19) show a slower migrating species of unknown origin.

Fig. 5D

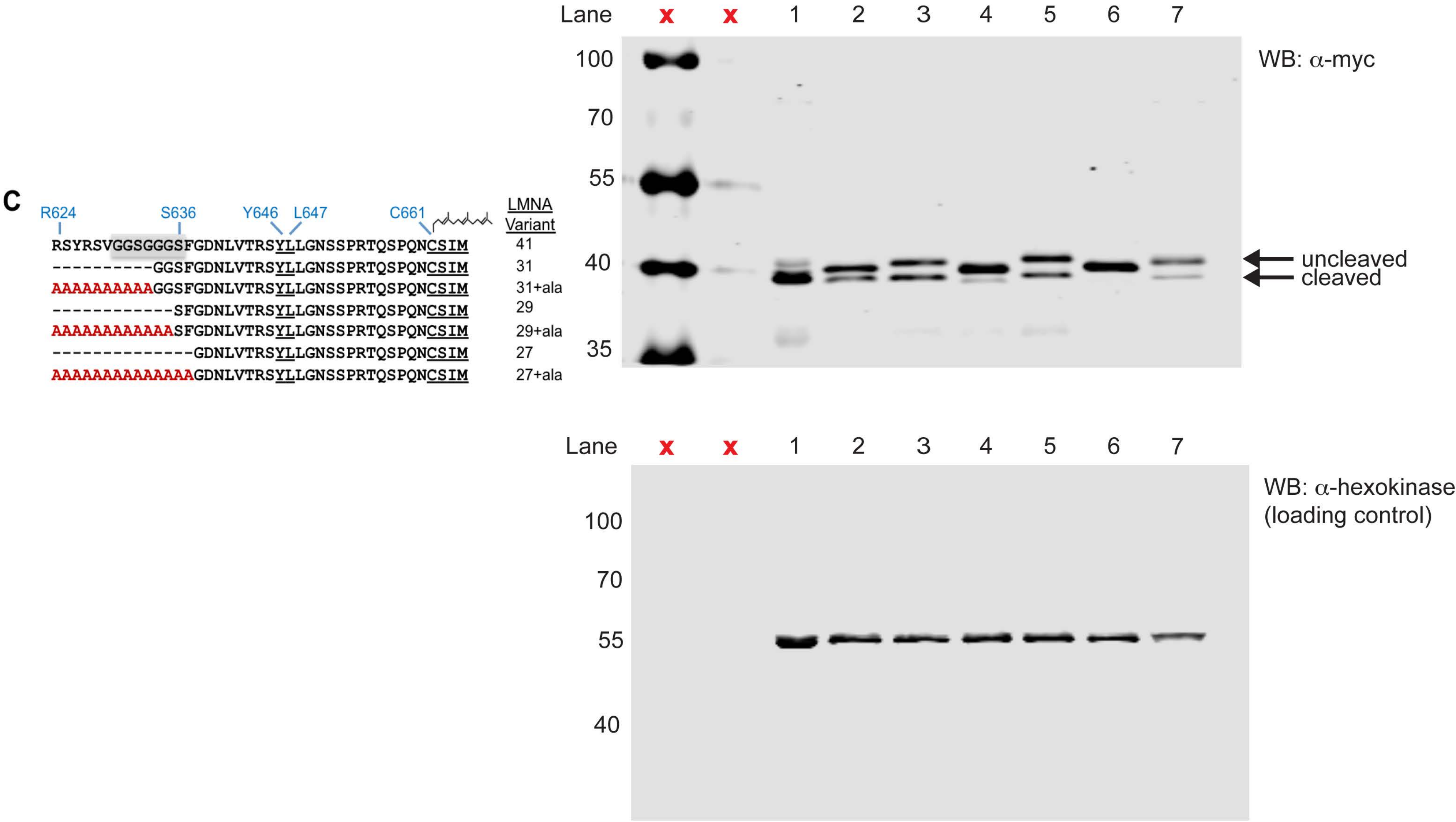

- SDS detergent lysates from strain SM6303 (*ste24Δ HA-ZMPSTE24*) transformed with myc-prelamin A plasmids were resolved by 10% SDS-PAGE, transferred to nitrocellulose (Biorad TransBlot Turbo), and proteins were detected using anti-myc monoclonal antibody and anti-hexokinase polyclonal antibody (described in M&Ms). Proteins were detected using Licor anti-mouse and anti-rabbit secondary antibodies and visualized using the Odyssey imaging platform (Licor). The longer alanine extensions (+14-+19) show a slower migrating species of unknown origin.

Fig. 6B

A

Y646 L647 C661

	LMNA Variant
RSYRSVGGSGGGSFGDNLVTRSYLLGNSSPRTQSPQNCSIM	**WT**
AAAAAAGGSGGGSFGDNLVTRSYLLGNSSPRTQSPQNCSIM	**624-629**
RSYRSVAAAAAAAFGDNLVTRSYLLGNSSPRTQSPQNCSIM	**630-636**
RSYRSVGGSGGGSAAAAAATRSYLLGNSSPRTQSPQNCSIM	**637-642**

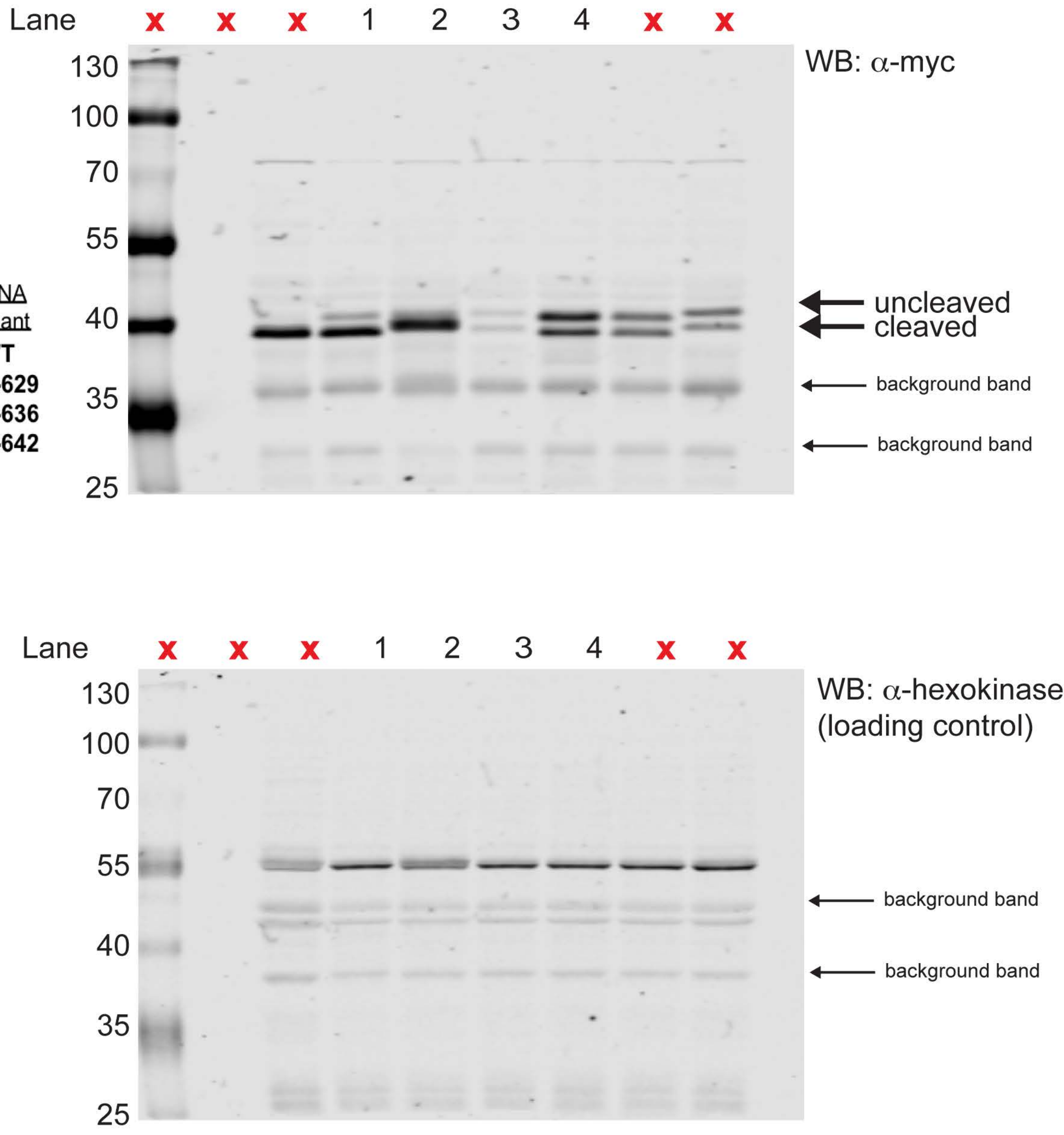

Fig. 6D

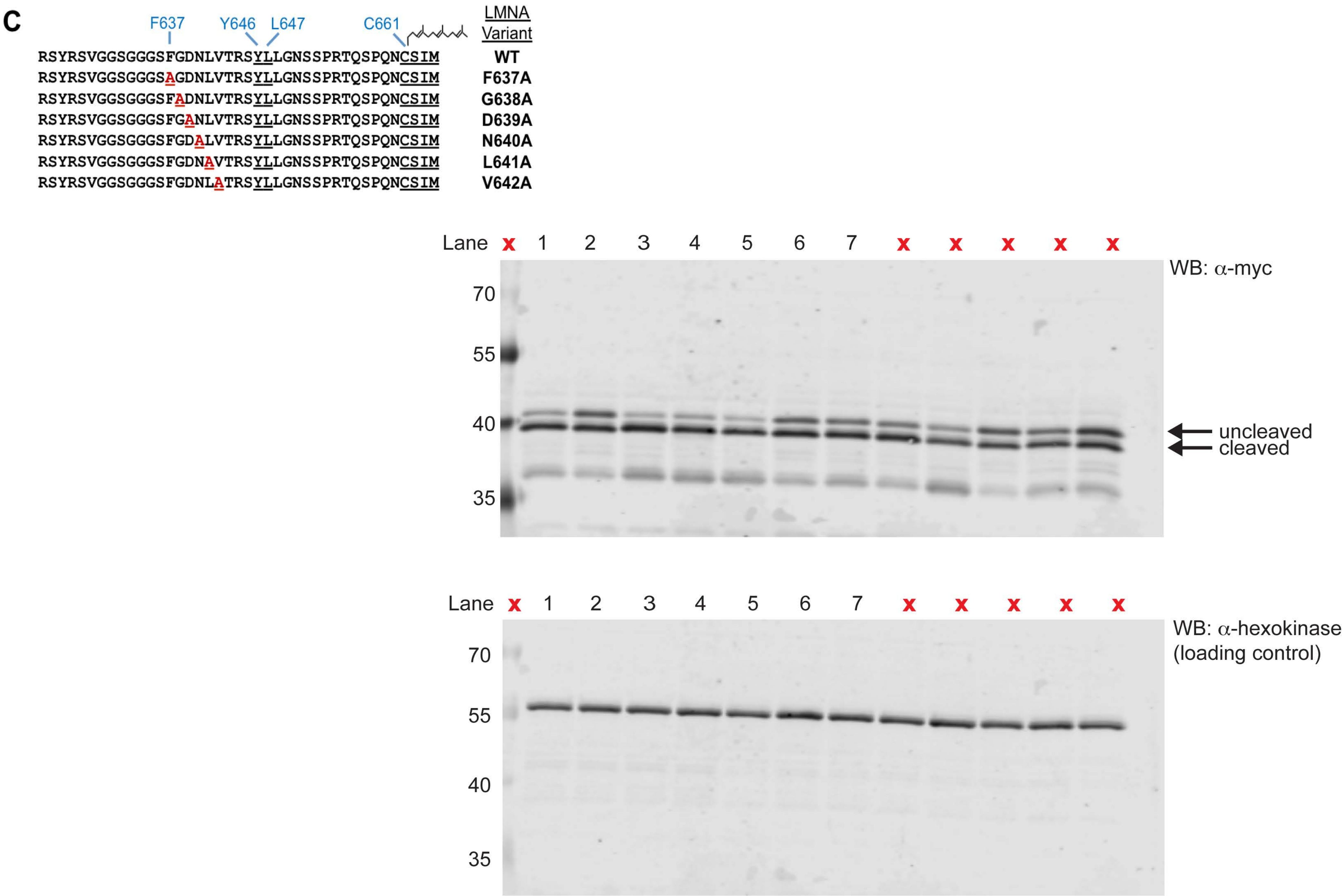
C
F637
Y646
L647
C661
LMNA Variant
RSYRSVGGSGGGSFGDNLVTRSYLLGNSSPRTQSPQNCSIM WT
RSYRSVGGSGGGSAGDNLVTRSYLLGNSSPRTQSPQNCSIM F637A
RSYRSVGGSGGGSFADNLVTRSYLLGNSSPRTQSPQNCSIM G638A
RSYRSVGGSGGGSFGANLVTRSYLLGNSSPRTQSPQNCSIM D639A
RSYRSVGGSGGGSFGDALVTRSYLLGNSSPRTQSPQNCSIM N640A
RSYRSVGGSGGGSFGDNAVTRSYLLGNSSPRTQSPQNCSIM L641A
RSYRSVGGSGGGSFGDNLATRSYLLGNSSPRTQSPQNCSIM V642A
Lane x 1 2 3 4 5 6 7 x x x x x
WB: α-myc
70
55
40
35
uncleaved
cleaved
Lane x 1 2 3 4 5 6 7 x x x x x
WB: α-hexokinase (loading control)
70
55
40
35

Supplemental Fig.S1

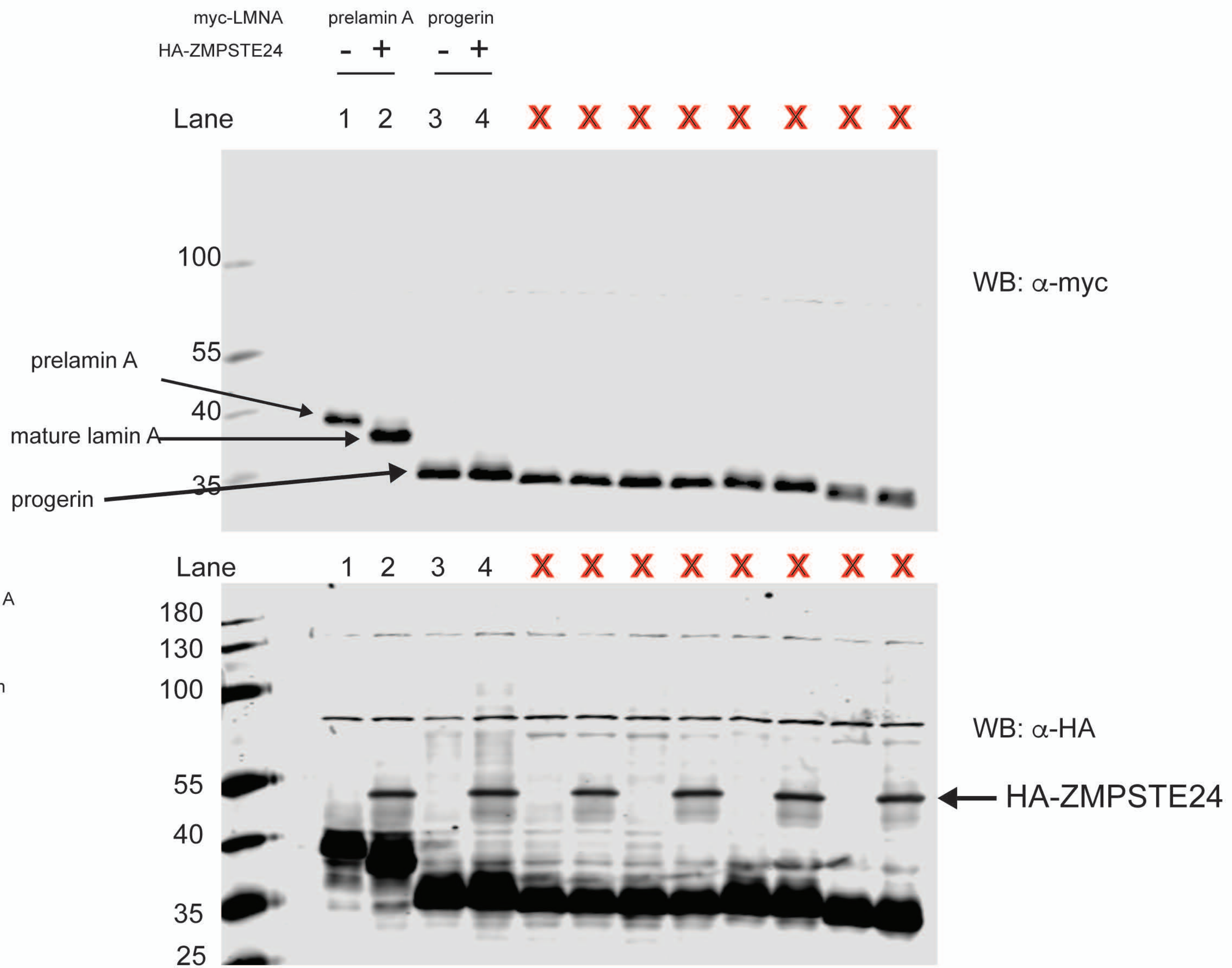

Lane

1 *ste24Δ* myc-prelamin A

2 *ste24Δ HA-ZMPSTE24* myc-prelamin A

3 *ste24Δ* myc-progerin

4 *ste24Δ HA-ZMPSTE24* myc-progerin

- SDS detergent lysates from strains SM4826 (*ste24Δ*) and SM6303 (*ste24Δ HA-ZMPSTE24*) transformed with myc-prelamin A (pSM3371) or myc-progerin (pSM3638) were resolved by 10% SDS-PAGE, transferred to nitrocellulose (Biorad TransBlot Turbo), and proteins were detected using anti-myc and anti-HA monoclonal antibodies (described in M&Ms). The myc blot was first developed using Licor anti-mouse secondary antibodies and visualized using the Odyssey imaging platform (Licor). After the myc blot was scanned, anti-HA rat monoclonal antibodies were used to detect HA-ZMPSTE24 (arrow).
